# Temporal niche dynamics of spreading native invertebrates underlie doubling of richness in pristine temperate streams

**DOI:** 10.1111/1365-2656.70005

**Published:** 2025-02-17

**Authors:** Michal Horsák, Michal Janáč, Marie Zhai, Jindřiška Bojková

**Affiliations:** ^1^ Department of Botany and Zoology, Faculty of Science Masaryk University Brno Czech Republic; ^2^ Institute of Vertebrate Biology Czech Academy of Sciences Brno Czech Republic

**Keywords:** aquatic invertebrates, increasing temperature, long‐term changes, niche filling, niche shift, streams, winners

## Abstract

1. While biodiversity loss is undeniably a global phenomenon, an increase in taxonomic richness has recently been reported from some ecosystems and spatial scales. A striking increase in abundance and/or species richness has been documented from temperate rivers over the last 25 years, with many of the expanding species (i.e. winners) being native species. However, the lack of repeatedly collected local environmental data prevents the exploration of their niche dynamics and also makes it difficult to distinguish between possible causes.
2. We fill this gap by using species occurrence data from 65 pristine Czech rivers sampled in 1997–2000 and 2015. The same methods were used for sampling macroinvertebrates and measuring environmental parameters in both periods. We selected 43 winners, defined as taxonomically validated and originally non‐rare native macroinvertebrate species whose occupancy increased by at least six sites between the time periods. We searched for consistent patterns of niche dynamics (i.e. stability, expansion and restriction) among species that might contribute most to the overall increase in species richness. Using several biological traits, we also compared the winners with the other 253 taxa collected to look for differences.
3. Analysis of the occurrence data showed that niche stability was by far the predominant pattern of the niche dynamics. This clearly indicates that the winners fill their original niches, with a limited contribution of niche shift or expansion, depending on the species. As no significant differences in either temperature preferences or the other biological traits were found between the winners and the other taxa, there is no unique set of functional traits that explain the success of the winners.
4. The observed mechanism of filling the original niche space by the spreading native species not only explains the increase in local species richness, but also contributes to support the hypothesis of a climate‐driven increase in ecosystem energy flow from a new perspective. The increased metabolism of the system may relax interspecific competition allowing it to carry more individuals and species, even without the need for an increase in nutrients and ecosystem recovery.

## Graphical abstract

Expanding native species of river macroinvertebrates mainly retained their original niches, with little evidence of niche shifts over the last 25 years. This suggests that species richness in pristine rivers increases due to enhanced ecosystem energy flow, relaxing interspecific competition and allowing it to carry more individuals and species.

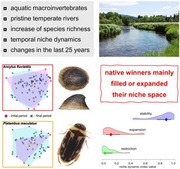

## 1 INTRODUCTION

In recent decades, massive changes in biodiversity have been observed at different spatial and temporal scales (Cardinale et al., 2012; Fanin et al., 2018; Hooper et al., 2012). The previously largely forecasted biodiversity decline is often debated, as it has turned out to be highly scale‐ and ecosystem‐dependent (Fanin et al., 2018; Hooper et al., 2012; Jarzyna & Jetz, 2018). On one hand, at large scales we are undoubtedly losing species. For example, rapid declines have been reported in many insects from the terrestrial realm (Conrad et al., 2006; Hallmann et al., 2017; Raven & Wagner, 2021). On the other hand, increases in biodiversity have been recorded in some assemblages at intermediate to small scales (Haase et al., 2019; Jarzyna & Jetz, 2018; Rumschlag et al., 2023). For example, the taxa richness and abundance of stream macroinvertebrate communities have often substantially increased over the last three decades (Haase et al., 2019; Haubrock et al., 2023; Pilotto et al., 2020). However, abundance change may differ among taxonomical and trophical groups (Powell et al., 2023), with its decrease recently found in North American rivers (Rumschlag et al., 2023).

Species richness increases when the species that spread to new locations (i.e. winners) outnumber the declining species (i.e. losers). In running waters, the winners are usually either opportunistic or thermophilic species (Bruno et al., 2019; Haase et al., 2019). There may also be other favourable life history, dispersal and habitat use characteristics (Bowler et al., 2021) or the reason for their success may be more complex and uncertain (Zhai et al., 2023). The important question arises whether the changes in distribution of winners are accompanied by changes in their environmental niches, for example, they occupy a wider or new set of conditions. Despite the general tendency of species to retain their realised niches in space and time (niche conservatism; Guisan et al., 2014), some species have capabilities to change their realised niches on a decadal timescale (Viana et al., 2023; Wiens et al., 2019), suggesting their fundamental niche space was broader than originally estimated.

Most knowledge on niche dynamics comes from the comparisons between native and non‐native ranges of invasive species (e.g. Guisan et al., 2014; Liu et al., 2020; Petitpierre et al., 2012; Wiens et al., 2019). However, many recently expanding species are of a native origin (Essl et al., 2019), and for those, the niche dynamics remain largely understudied (Lustenhouwer & Parker, 2022). In addition, most of the studies focus solely on macroclimatic niches and rarely included other coarser scale variables such as topography or geology (Viana et al., 2023). While this approach is highly relevant for large‐scale analyses (spatial and temporal) of species responses to climate change, it may not be suitable for the analyses of biodiversity patterns at fine regional scales (e.g. Wiens et al., 2019). At the regional scales, the role of non‐climatic environmental niche components is essential, yet largely neglected (Guisan et al., 2014), most probably due to the lack of available environmental data collected in time series, especially those that may change over time.

In this study, we attempt to fill this gap by examining the changes in non‐macroclimatic environmental niches of native winners during the last two decades. Further, by linking biological species traits to the niche change dynamics, we aim to understand the changes behind the current massive reorganisation of stream macroinvertebrate assemblages (Haase et al., 2019; Zhai et al., 2023). Our previous analyses of macroinvertebrates in temperate streams as close to natural as possible (hereafter referred to as pristine), collected repeatedly at the same sites, have documented a doubling in the number of species, while species decreasing their frequency of occurrence (i.e. ‘losers’) played only a negligible role (Zhai et al., 2023). The winners in this system were almost exclusively native and integral components of the assemblages (Zhai et al., 2023), in contrast to observed or predicted invasions by non‐native macroinvertebrates in streams due to warming (Daufresne et al., 2007; Domisch et al., 2011).

Our main objective was to explore niche dynamics of native winners in the environmentally well‐described system of pristine streams. We aim to look for consistent patterns of niche dynamics among the species that increase their occupancy the most and thus contribute the most to the overall increase in species richness of the studied assemblages (Figure S1a). High overlap in environmental spaces in the time periods of comparison and the extent of the study sites across a substantial part of the river continuum (i.e. from submontane brooks to low‐elevation rivers; Illies & Botosaneanu, 1963) allows the winners to track all main patterns of their niche dynamics (filling, expansion and shift; Wiens et al., 2019). Based on studies of macroclimatic niches, there is evidence that niche changes are likely limited within species' native ranges, for example, by biotic interactions (Wiens et al., 2019). Following this evidence, it is coherent to expect the native winners to show more conservatism in their environmental niches (niche filling) (H1a). However, given the scale of the recorded increase in richness and compositional changes in the assemblages (Zhai et al., 2023), we might equally expect their niche changed by expansion or shift (H1b). Further, we assume that most species expand or shift their niches towards upstream conditions compared to their environmental space in the initial period (H2). This hypothesis reflects observations for many macroinvertebrate and fish species in running waters that changed their distribution in upstream direction as a response to the ongoing climate change (e.g. Comte & Grenouillet, 2013; Daufresne et al., 2004; Haase et al., 2019). Our second objective was to search for functional traits that can explain the winners' success and observed niche change dynamics. Using a set of biological traits and thermal preferences used by Haase et al. (2019), we test whether winners differ in their biological traits from the other co‐occurring species (H3a) and/or are more thermophilic than the remaining taxa (H3b).

## 2 MATERIALS AND METHODS

### 2.1 Sampling site, biotic and environmental data

Data on aquatic macroinvertebrates and local environmental variables were collected from 65 streams of 2–7th stream Strahler order and altitudes of 150–800 m a.s.l. across all main river basins of the Czech Republic (map available in Zhai et al., 2023). The sites represented the least modified sites in terms of physical conditions, water quality, riparian zone and floodplain characteristics met the requirements for type‐specific reference conditions according to the Water Framework Directive, 2000/60/EC30 (Nijboer et al., 2004). The initial selection of these sites also considered their long‐term water quality development prior to the first sampling campaign (Kokeš et al., 2006), which resulted in the exclusion also of streams affected by acidification in the 1980s. This selection is the reason why both high‐elevation streams and large lowland rivers are poorly represented in the dataset as only a fraction of them fulfills the requirements to be considered pristine or at least near natural in the case of low‐altitude rivers.

The sampling of macroinvertebrates was done during spring (April–May) and autumn (September–October) in two periods: 1997–2000 (initial period of the first sampling campaign) and 2015 (final period, the last sampling), using the same standardised methodology of a multi‐habitat sampling method. For details on data collection and sample processing, see Zhai et al. (2023). Data from the spring and autumn samples of the same period were pooled for each site. The identification was done to species whenever possible, except for Diptera, some Oligochaeta and Coleoptera larvae that were identified to genera. Due to uncertainties of identifications in the initial period, all Chironomidae taxa had to be excluded from the dataset, and several species of Ephemeroptera, Plecoptera, Trichoptera and Oligochaeta had to be pooled to operational taxonomic units. This study neither require ethical approval, nor were any licences or permits needed to carry out the work.

Three criteria were applied to define the winners suitable for temporal niche change exploration: (a) unequivocal and reliable identification to species level, (b) occurrence frequency in the initial period of ≥6 (i.e. 10% frequency) and (c) increase in the occurrence frequency in the terminal period at least by six sites. From the total of 296 taxa, these three criteria were met by 43 of them (Table 1, Figure S1b). On the contrary, there were only five species that could be considered ‘losers’ based on the same numerical criteria. Because of such rarity of losers in our study system, we did not analyse their niche dynamics.

**TABLE 1:** List of 43 analysed winner species of aquatic macroinvertebrates.

Species (ordered by difference)	No. of sites with species occurrence (*N* = 65)				
	Higher taxa	Initial period	Final period	Difference	Both periods
*Centroptilum luteolum*	Ephemeroptera	10	50	40	9
*Platambus maculatus*	Coleoptera	10	48	38	8
*Pisidium personatum*	Bivalvia	9	41	32	8
*Pisidium casertanum*	Bivalvia	17	49	32	16
*Ephemera danica*	Ephemeroptera	24	51	27	24
*Nigrobaetis muticus*	Ephemeroptera	38	61	23	37
*Sialis fuliginosa*	Megaloptera	9	31	22	5
*Pisidium substruncatum*	Bivalvia	10	32	22	10
*Paraleptophlebia submarginata*	Ephemeroptera	25	46	21	22
*Ancylus fluviatilis*	Gastropoda	31	51	20	28
*Mystacides azureus*	Trichoptera	11	31	20	10
*Eiseniella tetraedra*	Clitellata	28	47	19	22
*Habrophlebia lauta*	Ephemeroptera	30	48	18	27
*Brachyptera risi*	Plecoptera	14	32	18	12
*Odontocerum albicorne*	Trichoptera	25	42	17	19
*Orectochilus villosus*	Coleoptera	16	33	17	16
*Leuctra nigra*	Plecoptera	7	22	15	5
*Lumbriculus variegatus*	Clitellata	12	26	14	5
*Sericostoma personatum*/*schneiderii*	Trichoptera	42	56	14	39
*Polycentropus flavomaculatus*	Trichoptera	26	40	14	25
*Nais alpina*	Clitellata	15	28	13	6
*Leuctra hippopus*	Plecoptera	26	39	13	24
*Nigrobaetis niger*	Ephemeroptera	8	21	13	7
*Ibisia marginata*	Diptera	26	38	12	23
*Silo pallipes*	Trichoptera	18	29	11	13
*Lepidostoma basale*	Trichoptera	15	26	11	13
*Cheumatopsyche lepida*	Trichoptera	7	18	11	7
*Dugesia gonocephala*	Tricladida	41	51	10	36
*Philopotamus montanus*	Trichoptera	9	18	9	5
*Ecdyonurus torrentis*	Ephemeroptera	28	37	9	24
*Hydropsyche siltalai*	Trichoptera	29	38	9	25
*Calopteryx virgo*	Odonata	7	16	9	5
*Oecismus monedula*	Trichoptera	7	16	9	6
*Habroleptoides confusa*	Ephemeroptera	41	50	9	40
*Athripsodes bilineatus*	Trichoptera	7	15	8	7
*Oulimnius tuberculatus*	Coleoptera	21	28	7	8
*Propapus volki*	Clitellata	29	36	7	22
*Agapetus ochripes*	Trichoptera	11	18	7	9
*Micrasema minimum*	Trichoptera	8	15	7	6
*Protonemura aestiva/auberti*	Plecoptera	6	13	7	5
*Nemoura cinerea*	Plecoptera	9	15	6	6
*Allogamus auricollis*	Trichoptera	15	21	6	14
*Athripsodes cinereus*	Trichoptera	7	13	6	7

To define the environmental niches of winners, we included three groups of environmental variables: (i) local environmental parameters, such as biological oxygen demand (BOD5), concentrations of total phosphorus (TP), nitrate‐nitrogen (NO_3_‐N), and ammonium‐nitrogen (NH_4_‐N), roughness of bed substrate (Phi) and share of riffles (Riff), all counted as means of the values measured six times during the year of macroinvertebrate sampling; (ii) proxies of land use (from a 250 m wide river side strip across 15 km of stream network above the site), that is, areas of unfavourable surfaces (Un_surf) and forested area (Fore), computed based on the information from the CORINE Land Cover system (see Zhai et al., 2023 for details on how the parameters were measured); and (iii) climatic variables, such as mean air temperatures in January and July (T_Jan, T_Jul), and annual precipitation (Prec), which were calculated based on values of daily averages in the year of sampling and two consecutive years prior to sampling, using gridded data provided by the Czech Hydrometeorological Institute (Polášek et al., 2017).

### 2.2 Statistical analyses

To describe temporal niche dynamics of the winners, we have used the COUE (centroid shift, overlap, expansion and unfilling) framework metrics (Guisan et al., 2014). The COUE metrics are calculated using species occurrence densities in a gridded environmental space (Broennimann et al., 2012). First, we expressed the main gradients in our environmental data using Principal Component Analysis (PCA). We observed a shift in air temperatures between the initial and final periods, corresponding to an average warming of 2.51 ± 0.47 °C in January and 2.40 ± 1.17 °C in July. However, this unidirectional systematic shift resulted in almost non‐overlapping environmental spaces (Figure S2), which prevented a reliable examination of the environmental niche differences (Guisan et al., 2014). Therefore, air temperatures had to be excluded from the set of environmental variables defining the niche space. However, as the increase in air temperature is likely to be tightly correlated with some, but certainly milder, increase in water temperature, we projected the air temperature data onto the resulting PCA space (using the *vegan* function *envfit*; tested based on 4999 permutations).

Based on the occurrence densities of the winners in the environmental space, defined by the first two PCA axes, for each winner, we calculated the dynamic niche indices: niche stability, that is, the proportion of the niche in the final period shared with the initial period niche; niche restriction, that is, the proportion of the initial period niche that does not overlap with the final period niche (which corresponds to niche unfilling in Broennimann et al., 2012); and niche expansion, that is, the proportion of the final period niche non‐overlapping with the initial period niche. To evaluate the robustness of these niche dynamic indices, we applied the leave‐one‐out jack‐knife procedure (i.e. for each species, niche dynamic indexes were recalculated in 1000 iterations, with one of the species observations randomly omitted in each iteration). Further, we also calculated the classical Schoener's D niche overlap value (Schoener, 1968) and tested the significance of detected niche overlap against 1000 randomised assemblages. The niche similarity test (Warren et al., 2008) was performed for each winner to test the hypothesis of niche conservatism (the null hypothesis states that the niche overlap D value between the initial and final periods is not different from those generated by 1000 randomised assemblages). In addition, for each winner, we calculated the number of sites occupied in the initial period and the number of sites newly colonised in the final period. All these six niche‐related parameters were used in a cluster analysis (Ward's method, Euclidean distance) to simplify the description of niche dynamic results by defining homogeneous categories of winners with consistent niche change characteristics. Differences in each of the six parameters among the winner categories were tested using analysis of variance (ANOVA, applied on arcsin‐transformed data for niche overlap, expansion, restriction and stability), followed by Tukey HSD post hoc tests. Finally, we assessed the shift in the positions of species centroids between the periods along the PCA axes to quantify the upstream and downstream movement (first PCA axis) or the movement along the sediment grain‐size spectrum (second axis; for definition of the axes, see the Results). These two trends in shifts of winner centroid scores along the two PCA axes were tested using paired *t*‐tests.

We used fuzzy correspondence analysis (FCA; Chevenet et al., 1994) and permutational multivariate analysis of variance (PERMANOVA; Anderson, 2001) to determine whether the winners possessed biological traits that distinguished them from the other species. Each of the 296 taxa included in the analysis was characterised by seven biological traits describing body size, feeding behaviour, locomotion, respiration, dispersal strategy and potential and life cycle (Table S1). Trait data were taken from published sources (Sarremejane et al., 2020; Tachet et al., 2000) and online databases (Polášek et al., 2017; Schmidt‐Kloiber & Hering, 2015). Traits with 29 modalities were coded using the fuzzy approach to assign the affinity of species to a particular trait modality (Chevenet et al., 1994). FCA conducted on the trait‐by‐species matrix was used to ordinate species in the functional trait space. PERMANOVA was used to determine the differences in the coordinates of the functional trait space (on all 22 FCA axes) between the winners and the other species from the initial period, in order to explore whether there had been a set of traits predicting winners' success, and the differences among the winner categories. We also tested whether winners were more warm‐adapted than the other species using the approach of Haase et al. (2019), to associate the expansion of winners with warming. This approach defines the temperature niche of species based on their affinities to river zones (Illies & Botosaneanu, 1963). For more details and literature, see Haase et al. (2019). The data were available for all winners and for 232 of the 253 remaining taxa. Mann–Whitney test and Kruskal–Wallis test were used to test the differences in temperature preference between the winners and the other species from the initial period, and among the winner categories, respectively.

All statistical analyses were conducted using R 4.1.2 (R Core Team, 2021), using the *ade4* (Chessel et al., 2004), *ecospat* (Broennimann et al., 2023) and *vegan* (Oksanen et al., 2020) libraries.

## 3 RESULTS

**FIGURE 1:**
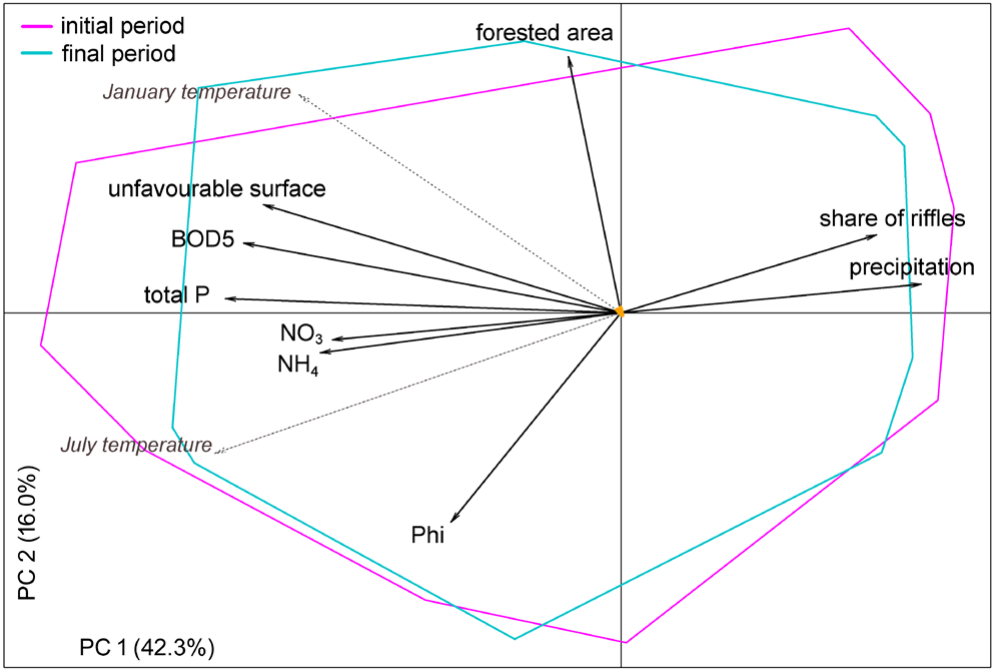
Available environmental spaces in initial and final periods expressed by the first two PCA axes based on environmental variables used for the calculation of niche dynamic indices. The orange arrow shows the centroid shift. July and January air temperatures, given in grey and italics, were only projected into the resulting ordination diagram. The environmental variables with the highest load on PCA 1 roughly reflect the environmental changes along the river continuum.

The first two axes of the PCA explained 58.3% of the total variability (Figure 1). The first axis (42.3%) was associated negatively mainly with total P, BOD5, unfavourable surfaces, NO_3_ and positively mainly with annual precipitation, which all corresponded to the main environmental changes along the river continuum. The second axis (16.0%) was associated positively mostly with forested area and negatively with Phi, structuring the sites from small grain‐size streams with less forested catchment areas to the opposite. The projections of January and June air temperatures were both negatively associated with the first axis (Figure 1). However, a significant load (*p* = 0.001) was found only for July air temperature, with regression coefficients of −0.89 (PC 1) and −0.45 (PC 2), and adj. *R* ^2^ of 10.5%. There was a large overlap between the available environmental spaces of the study periods (*D* = 0.75; Figure 1), with an almost negligible shift in the environmental centroid between them (0.27% and 0.77% of the gradient length on the first and second PCA axis, respectively), enabling unambiguous interpretation of niche dynamic changes.

**FIGURE 2:**
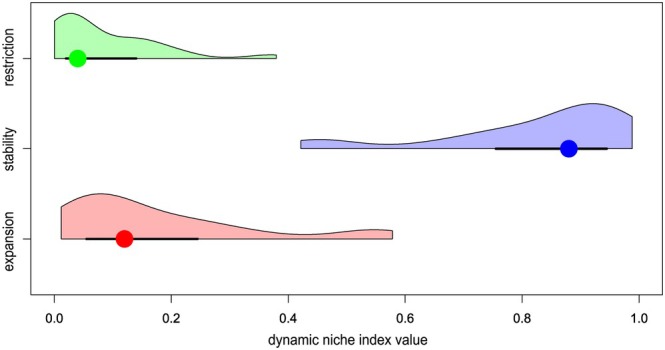
Distribution of the three dynamic niche indices (niche expansion, stability and restriction) among the 43 winner species. Point = median, wide line = interquartile range and thin line = range. Areas under each curve represent idealised density distribution.

**FIGURE 3:**
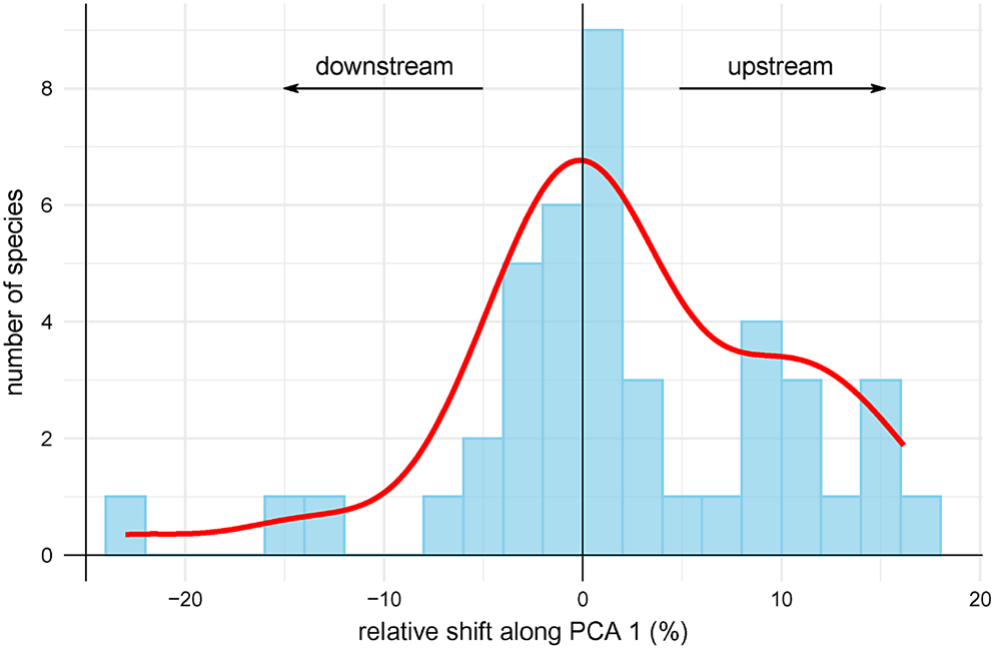
The distribution of winners according to the shift in their niche centroids along the first PCA axis, corresponding to the river continuum. The shift was standardised for the gradient range (see Table S2).

The temporal niche dynamics of winners showed mostly stability (median = 0.88), while niche expansion (median = 0.12) and restriction (median = 0.04) were less frequent (Figure 2), with the same results obtained after the validation (Figure S3). Niche similarity tests were significant in 25 out of 43 winner species, suggesting niche conservatism for more than half of the winners (Table S2). Eleven and five of the winners shifted their centroid positions by more than 10% of the first and second environmental gradient length, respectively (Table S2). Although eight of those 11 winners expressed upstream shifts compared to the remaining three that shifted downstream (Figure 3), the overall numbers of upstream and downstream changes were less uneven (26 vs 17 species, respectively), showing a statistically not significant difference (paired *t*‐test, df = 42, *p* = 0.122). Likewise, there was no inter‐period difference in winner centroid scores along the second PCA axis (paired *t*‐test, df = 42, *p* = 0.861).

**FIGURE 4:**
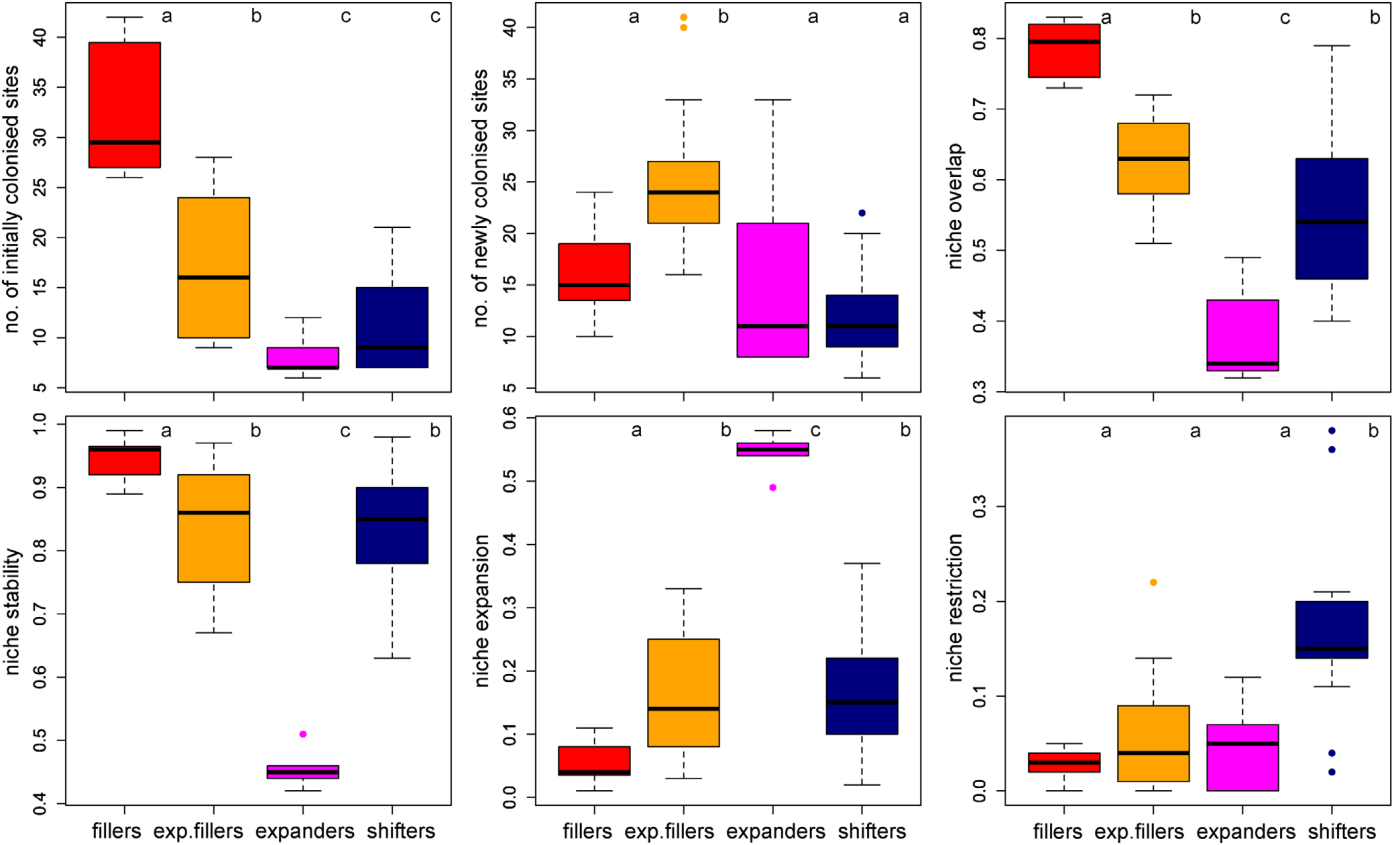
Variation in six niche‐related parameters among four categories of winners. The central line of each box refers to the median value, box height to the interquartile range, whiskers to the non‐outlier range (i.e. 1.5 times the interquartile range) and small circles to outliers. Significant differences at *p* < 0.05, based on ANOVA and Tukey HSD post hoc tests, among the categories are shown by letters (same letter means no significant difference, while different letters refer to significant differences between boxes).

**FIGURE 5:**
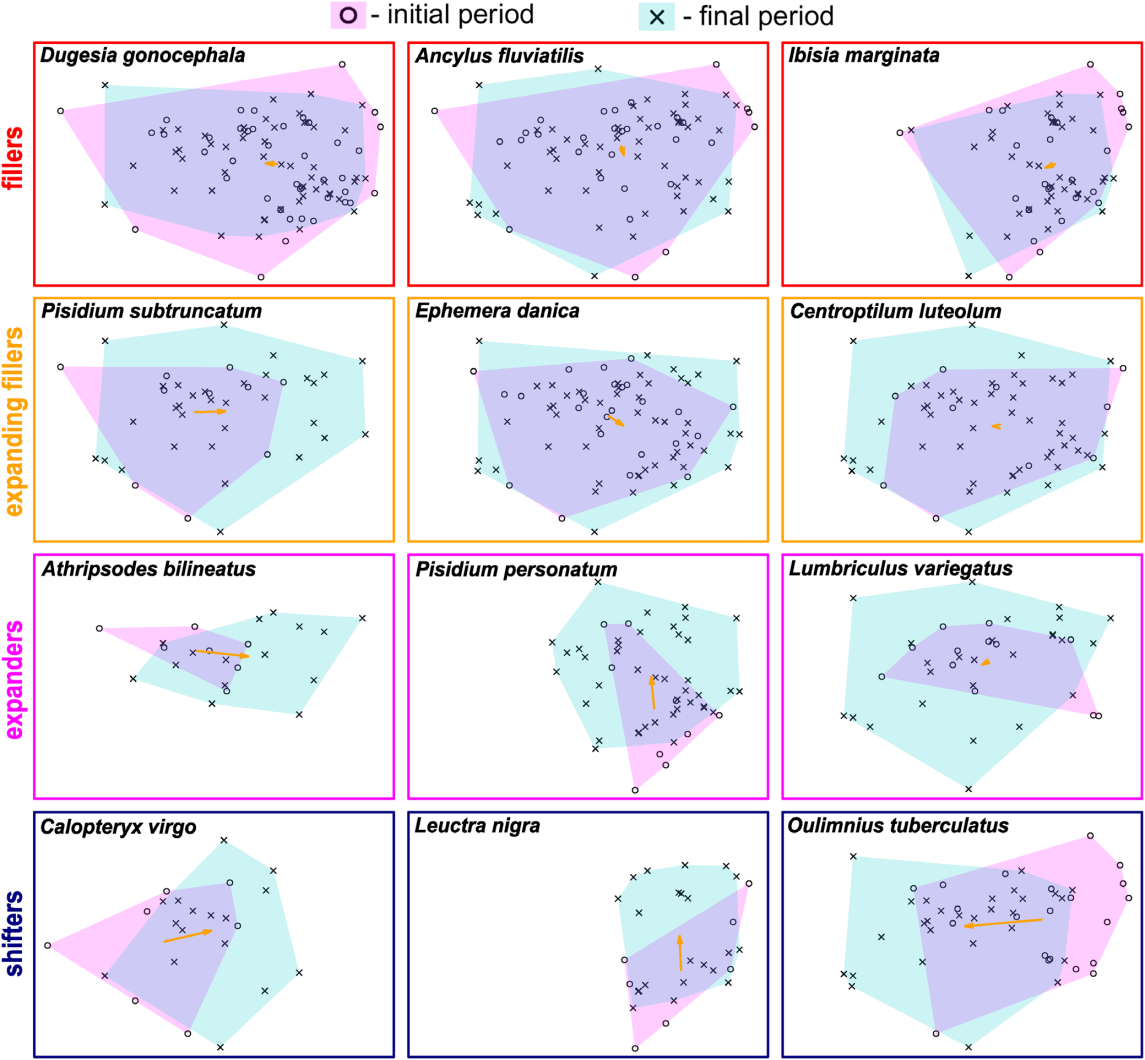
Examples of winner species representing four main patterns of their environmental niche shifts from the initial to final period. The orange arrow shows centroid shift. See Figure 1 for further details on environmental niche space and main environmental gradients.

Based on cluster analysis, the winners could be sorted into four main categories according to their niche‐related parameters (Figure 4 and Figure S4). (1) The winners having a significantly higher niche overlap and stability than the other categories (Figures 4 and 5) filled their niches by new colonisation of sites within their initially occupied niches (‘fillers’; 12 spp., i.e. 28%). They expressed a significant niche conservatism (niche similarity tests, all *p* < 0.03, see Table S2 for exact *p* values) and fell out of the other three groups. (2) The winners that showed high values of niche overlap and stability, with nine of them also expressing significant niche conservatism (all *p* < 0.05, Table S2), not only filled their initial niches but also slightly expanded their niches (‘expanding fillers’; 13 spp., i.e. 30%). (3) The winners being relatively rare in the initial period expanded their niche significantly more than species of other categories (Figures 4 and 5). None of these winners (‘expanders’; 5 spp., i.e. 12%) showed significant niche conservatism (all *p* > 0.05, Table S2). (4) Finally, the winners that typically occupied relatively small numbers of both initially and newly occupied sites shifted their niches (‘shifters’; 13 spp., i.e. 30%) by a combination of slight niche expansion and significant niche restriction (Figure 5). Four of them showed significant niche conservatism (all *p* < 0.03, Table S2). For changes in the environmental niche space of all species, see Figure S5a‐c.

**FIGURE 6:**
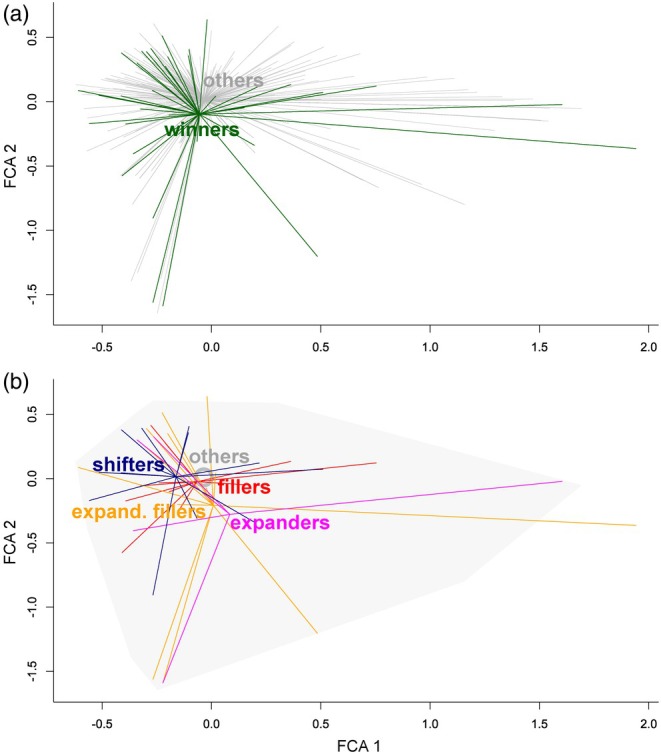
(a) Position of winners and other species along the first two axes of FCA representing the functional trait space along these axes. Each species is represented by a line directing to a respective category centroid; (b) position of the four winner categories along the first two axes of FCA representing the functional trait space. Each winner species is represented by a line directing to a respective category centroid. Grey area represents space covered by other species, with grey circle representing their centroid. Note that tests of the differences between these categories considered all 22 existing axes.

**FIGURE 7:**
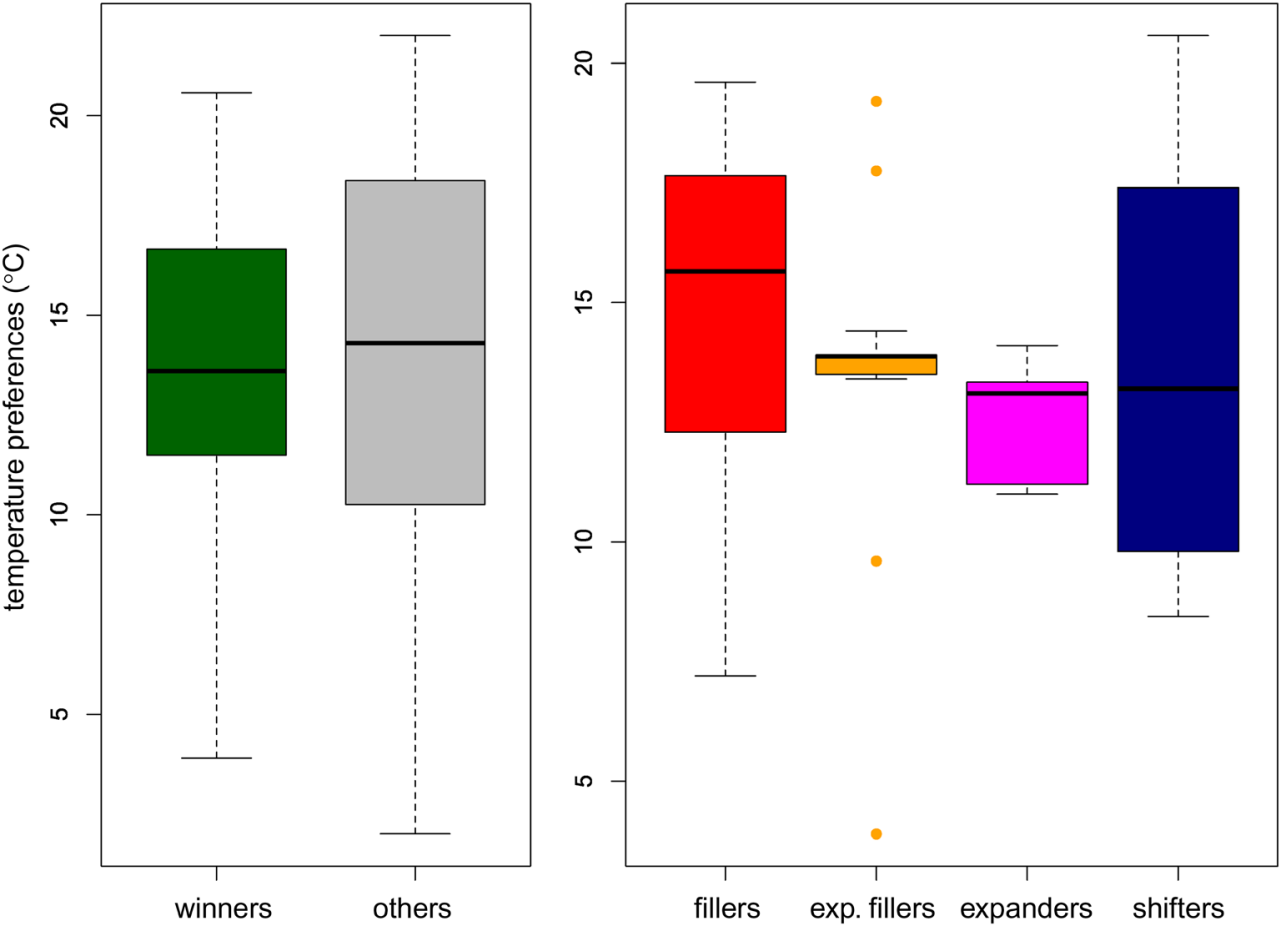
Temperature preferences of winners and other species (left panel) and of four winner categories (right panel). There were no statistically significant differences (Mann–Whitney and Kruskal–Wallis test, *p* = 0.565 and 0.617). For details on boxplots, see the caption for Figure 4.

The positions of winners in the functional trait space neither significantly differed from those of the other species (PERMANOVA, df = 1, 294, *p* = 0.595; Figure 6a), nor did their positions among the categories of winners (PERMANOVA, df = 3,39, *p* = 0.617; Figure 6b). Temperature preferences also did not differ significantly between the winners and the other species (Mann–Whitney *U* test, *N* = 275, *p* = 0.565), nor among the winner categories (Kruskal–Wallis test, *N* = 43, *p* = 0.860, Figure 7).

## 4 DISCUSSION

Until now, the studies from running waters have explored only the changes in macroclimatic niches of invasive species (Šidagytė‐Copilas & Copilaș‐Ciocianu, 2023; Torres et al., 2018), mainly fish (Gibson‐Reinemer & Rahel, 2015; Kirk & Rahel, 2022). To the best of our knowledge, this is the first study to analyse the niche dynamics of stream macroinvertebrates using non‐climatic variables, trying to decipher the causes of increasing taxonomic richness (Zhai et al., 2023) and species occupancy. We could do so by having unique data based on high‐resolution taxa identification in both sampling periods and accompanied by direct measurements of environmental conditions, including water chemistry under the same protocol. A large overlap between the available environmental spaces for both periods enabled us to test species temporal niche dynamics at finer spatial resolution (Guisan et al., 2014).

As our sites represent the most natural streams possible, the observed changes can be primarily associated with the ongoing climate warming and its effects on freshwater ecosystems (Woodward et al., 2010). The results show that realised niches can be dynamic and may reflect changes on a decadal scale, not only in response to biological invasions (Viana et al., 2023). We have confirmed our hypothesis (H1a) on the prevailing niche conservatism (in 58% of all species) among different pathways of niche dynamics. On the contrary, many species significantly expanded their niches, suggesting the existence of some mechanisms restricting their realised niches in the initial period (Soberón & Arroyo‐Peña, 2017). We also detected niche shifts and expansions mainly to up‐ and downstream conditions. Unexpectedly, we did not find any support for the winners' success promoted by their functional traits and known temperature preferences, nor was there any association of these traits and niche change characteristics.

### 4.1 Environmental niche changes

We confirmed our hypothesis (H1a) that niche filling would be significant in most winners. About 58% of all winners had a significant niche overlap, regardless of other niche change characteristics. About 30% of all winners colonised sites strictly within their original niche space (‘fillers’, e.g. the common species: tricladid *Dugesia gonocephala* and gastropod *Ancylus fluviatilis*). Nine more winners with low initial frequencies (‘expanding fillers’) significantly increased their frequencies in the final period, their original niches being nested within the new niche space. These species were, in fact, the most successful of all winners (e.g. mayflies *Centroptilum luteolum* and *Ephemera danica*). Even among the species showing some clear shifts in their niches, four of them still expressed significant niche stability. This could be expected because niche conservatism appears to be a universal mechanism even for species in their non‐native ranges (Aravind et al., 2022; Liu et al., 2020; but see Bates & Bertelsmeier, 2021) where they are not limited by biotic interactions (Wiens et al., 2019).

However, despite the predominance of niche filling, more than half of the winners shifted (30% of all winners) or expanded (12%) their environmental niches. As expected, most of these changes occurred along the first PCA axis, which, based on a combination of environmental variables associated with this axis, can be interpreted as a change from colder upstream to warmer downstream sections, that is, along the river continuum. In agreement with our H2, more directional expansion or shift towards the upper sections was found (Comte & Grenouillet, 2013; Freeman et al., 2018; Haase et al., 2019). Given the large temperature changes (Zhai et al., 2023), the predominant upstream shift was expected. Upstream niche changes could be the result of relaxed limitations by winter temperatures. Within this interpretation, Haase et al. (2019) reported a proportional increase of downstream‐adapted taxa in central European small low mountain streams. In our study, eight winners shifted their niches to upstream conditions by more than 10% of their original range on the respective gradient. For example, the diving beetle *Platambus maculatus* notably expanded upstream and the damselfly *Calopteryx virgo*, recently increasing in Central Europe (Bowler et al., 2021), clearly shifted upstream. Marked expansion towards brooks was found in several trichopterans, such as *Athripsodes bilineatus* and *Cheumatopsyche lepida*, the species preferring low‐rhithral and epipotamal streams (Graf et al., 2008). Further detection of upstream shifts in our data might have been reduced due to omitting the large rivers of low elevations, as these were under heavy anthropogenic influence, thus not included in the set of our monitored sites.

Unexpectedly, some winners showed chiefly downstream shifts and expansions, three of them exceeding 10% of the gradient length. The riffle beetle *Oulimnius tuberculatus* showed the most pronounced downstream shift (23%) and the stonefly *Brachyptera risi* expanded to the lower stream sections. Tison‐Rosebery et al. (2022) reported a long‐term increase in riffle beetles in French streams since the 1990s, and *B*. *risi* was found to be the only native stonefly species currently increasing in Czech streams since the 1950s (Bojková et al., 2012). The downstream niche shifts might be the result of an indirect effect of temperature conditions, relaxing mechanisms and conditions making the pre‐warming fundamental niche of these species more restricted (Soberón & Arroyo‐Peña, 2017). We recorded an average increase in mean air temperatures of ca 2.5°C during the sampling period, which is consistent with long‐term trends in stream water temperature in the Czech Republic, which showed the average increase of 0.04 °C per year in the period 1980–2006 (Novický et al., 2009). Such an increase in temperature can promote metabolic activity and physiological rates of species, resulting in altered respiration (Hamburger & Dall, 1990), growth (Janssens et al., 2015; Pöckl, 1992) or development (Gillooly et al., 2002; Pöckl, 1992). Given that the increase in species richness was associated with a massive increase in abundance (Figure S1), we assume that the observed changes can be related to the proposed mechanism of the increase in ecosystem energy flow (Arora et al., 2016; Haase et al., 2019), possibly also leading to a higher complexity in food webs (Van Looy et al., 2016). The increase in energy and resources may allow species to colonise sites occupied by stronger competitors, as the increased carrying capacity may relax competition induced by niche overlap between species (Krebs, 2014; Pastore et al., 2021). Nordberg and Schwarzkopf (2019) reported that reduced competition may facilitate generalist species to expand their niches at both the microhabitat and landscape level. This would apply to many of our winner species, clearly those common and spanning across most of the river continuum (i.e. the main environmental gradient in river systems) already during the initial period. Many of the winner species were already widespread in the initial period or even earlier (e.g. *Ancylus fluviatilis*, *Platambus maculatus* and *Ephemera danica*) and some have also been generalists, often inhabiting other aquatic habitats (e.g. *Pisidium subtruncatum* and *Eiseniella tetraedra*).

Further, some upstream adapted ‘shifters’ and ‘expanders’ remained constricted in the upper zone of the river continuum but shifted/expanded along the second PCA axis. For example, the upstream adapted plecopteran *Leuctra nigra* and bivalve *Pisidium personatum* shifted/expanded towards the sites with higher proportions of forest area in the catchment and rougher sediment. Although we are far from understanding these shifts, an explanation can be proposed for some species. Since *Pisidium* species feed on very fine organic particles and bacteria (Lopez & Holopainen, 1987), we can hypothesise that thermally promoted bacterial activity and growth may provide a previously absent or limited food source. However, direct measurements of bacterial growth or data on FPOM content in both sampling periods would be necessary to test this assumption. Buffering of summer temperature extremes by increasing forest cover can be an alternative mechanism, which might be valid for some cold‐stenotherm insects. In general, all these changes leading to the reduction of competition or limiting factors could modify species’ realised niches, clearly in expanding species, by unfolding previously restricted fundamental niches (Soberón & Arroyo‐Peña, 2017).

### 4.2 Species traits of winners

There is ample evidence of recent functional changes in benthic invertebrate communities (e.g. Van Looy et al., 2016), often related to climatic processes (Floury et al., 2018), anthropogenic stressors (Mondy & Usseglio‐Polatera, 2014) and biological invasions (Latli et al., 2017). As most changes in abundance and taxonomic richness at our sites were caused by native species (Zhai et al., 2023), this raised the question of the extent to which winners might represent a specific cocktail of biological traits. In our data, we could not detect a common combination of traits among winners that distinguished them from other species and would therefore explain their success. This might be related to the fact that the winners were natural components of the communities, spanning the whole environmental space area, with their trait diversity highly overlapping with the trait space occupied by all other species recorded in the initial period. There is some evidence that good dispersal abilities might be one of the favourable species attributes of winners (Bowler et al., 2021), particularly under ongoing climate change (De Bie et al., 2012). Indeed, in our study, most of the winners were insects with active flying adults (ephemeropterans, coleopterans, dipterans and trichopterans). However, there were also some high‐scoring winners among the passively dispersing taxa (flatworms, molluscs and clitellates), despite a strong disproportion in favour of active dispersers in the species pool. Therefore, it seems that dispersal ability is not the main promoter of our winners' success.

An increase in warm‐stenothermic and/or eurythermic taxa has been repeatedly documented in stream macroinvertebrate assemblages (Bruno et al., 2019; Domisch et al., 2013; Haase et al., 2019), referred to as thermophilisation. However, our analyses did not detect differences in thermal preference between the winners and other species. It is possible that thermal preferences are in fact only a very rough estimate of species' temperature niches, showing patterns only on a larger spatial scale. Field observations give only limited information about fundamental thermal niches of species, while proper laboratory measurements of thermal niches are scarce for aquatic macroinvertebrates (e.g. Calosi et al., 2008; Carbonell et al., 2024; Dallas, 2016). However, given the low levels of niche restrictions in our study, the current thermal conditions were clearly within the thermal optima of winners.

Several mechanisms have been considered to promote the observed increase in species richness and abundance of stream macroinvertebrates. Among these, the improvement of water quality (e.g. Van Looy et al., 2016; Vaughan & Ormerod, 2012) and changes in nutrient concentration and stoichiometry have been identified as the most important (e.g. Beck et al., 2023; Friberg et al., 2010; McCormick et al., 2004). However, it is unlikely that these processes play a significant role in our study system. Our data come from the least affected sections of the preserved Czech rivers (Zhai et al., 2023), which have not been affected by pollution or acidification prior to the initial sampling campaign (Kokeš et al., 2006). At these sites, water quality was carefully monitored and did not show significant, consistent changes in any of the trophic status indicators (Table S3), that is, total phosphorus, nitrates and BOD5 (Zhai et al., 2023). However, ecosystem energy flow may have changed even without a change in nutrient load, as discussed above. Although it has been suggested that increasing temperatures boost the metabolism of the whole system (Arora et al., 2016; Haase et al., 2019), direct measurements for such an increase in energy flow are still lacking, clearly due to the absence of any pre‐warming data.

Our results can add qualitatively new evidence to the increased energy flow hypothesis, as (i) there was no obvious change in nutrient load documented by direct water quality measurements in both periods studied, (ii) the pristine character of the study sites did not lead to changes in channel characteristics and in‐stream habitats, (iii) the massive increase in total abundance was observed using the same sampling method and (iv) the doubling of species richness was almost exclusively due to native species that were already common in the regional species pool in the pre‐warming period. As a result, the majority of widespread species were present wherever they could be, and in many cases, they even colonised new, previously unoccupied environments, as in the case of species that were restricted by low temperatures and have spread upstream. A combination of all these patterns of niche change could explain such a profound change in river diversity without a significant spread of new invaders. It leads to a massive restructuring of stream assemblages and has the potential to modulate the complexity of the food web and biotic interactions. However, we may only be documenting the initial phases of this process, which is still buffered by the resilience of the ecosystem.

## AUTHOR CONTRIBUTIONS

Michal Horsák and Michal Janáč designed the study; Michal Horsák, Marie Zhai and Michal Janáč wrote the first draft; and Jindřiška Bojková curated and interpreted the data. All authors edited the manuscript.

## CONFLICT OF INTEREST STATEMENT

None declared.

## STATEMENT ON INCLUSION

Data were generated in cooperation with the administrations of river catchments, and the results will help to make decisions supporting biodiversity and good ecological conditions of the river ecosystems.

## Supporting information


**Table S1.** List of biological traits used to describe functional trait space of 296 species present in the initial period.
**Table S2.** Niche overlap (D), *p*‐value of the niche similarity test (*p*‐D) and three dynamic niche indices (niche expansion, stability and restriction) for winners belonging to four categories: ExFil, expanding fillers; Fill, fillers; Exp, expanders; Shif, shifters.
**Table S3.** Descriptive statistics of the used environmental variables, separately for each period.
**Figure S1.** (A) total abundance (i.e. summed across all species) and taxa richness at the 65 study sites in the initial and final periods calculated for (a) all taxa, (b) winner species only and (c) other taxa only. For details on boxplots see the caption for Figure 4. (B) variation in changes of species abundance (summed across all sites) and occurrence showed across all species, winners only and other species only.
**Figure S2.** Available environmental spaces in initial and final periods expressed by the first two PCA axes based on all environmental variables including air temperature data.
**Figure S3.** Position of the original observed value of three niche dynamic indexes (niche expansion, stability and restriction) within a range of 1000 values obtained by leave‐one‐out jack‐knife procedure.
**Figure S4.** Dendrogram visualising the results of cluster analysis of six niche‐related parameters (Ward's method, *k* = 4), resulting in four winner categories marked by coloured rectangles.
**Figure S5a.** Environmental niche shifts from initial to final period for winner species classified as fillers and expanders.
**Figure S5b.** Environmental niche shifts from initial to final period for winner species classified as expanding fillers.
**Figure S5c.** Environmental niche shifts from initial to final period for winner species classified as shifters.

## Data Availability

Data are available from the Dryad Digital Repository: https://doi.org/10.5061/dryad.zpc866tk0 (Horsák et al., 2025).
