# Supplementary material for: Temporal niche dynamics of spreading native invertebrates underlie doubling of richness in pristine temperate streams
Source: J Anim Ecol. 2025 Feb 17;94(4):693–705. doi: 10.1111/1365-2656.70005 (PMC11962250; doi:10.1111/1365-2656.70005)
Supplement: Supplementary file 1 — Table S1. List of biological traits used to describe functional trait space of 296 species present in the initial period. Table S2. Niche overlap (D), p‐value of the niche similarity test (p‐D) and three dynamic niche indices (niche expansion, stability and restriction) for winners belonging to four categories: ExFil, expanding fillers; Fill, fillers; Exp, expanders; Shif, shifters. Table S3. Descriptive statistics of the used environmental variables, separately for each period. Figure S1. (A) total abundance (i.e. summed across all species) and taxa richness at the 65 study sites in the initial and final periods calculated for (a) all taxa, (b) winner species only and (c) other taxa only. For details on boxplots see the caption for Figure 4. (B) variation in changes of species abundance (summed across all sites) and occurrence showed across all species, winners only and other species only. Figure S2. Available environmental spaces in initial and final periods expressed by the first two PCA axes based on all environmental variables including air temperature data. Figure S3. Position of the original observed value of three niche dynamic indexes (niche expansion, stability and restriction) within a range of 1000 values obtained by leave‐one‐out jack‐knife procedure. Figure S4. Dendrogram visualising the results of cluster analysis of six niche‐related parameters (Ward's method, k = 4), resulting in four winner categories marked by coloured rectangles. Figure S5a. Environmental niche shifts from initial to final period for winner species classified as fillers and expanders. Figure S5b. Environmental niche shifts from initial to final period for winner species classified as expanding fillers. Figure S5c. Environmental niche shifts from initial to final period for winner species classified as shifters. [file JANE-94-693-s001.pdf]

Supplementary Materials for:

Temporal niche dynamics of spreading native invertebrates underlie doubling of richness in pristine temperate streams

Michal Horsák*, Michal Janáč, Marie Zhai, Jindřiška Bojková

*correspondence: horsak@sci.muni.cz

This PDF file includes:

Tables S1 to S3

Figures S1 to S5

Table S1: List of biological traits used to describe functional trait space of 296 species present in the initial period.

biological traits	data fill (%)	modality				
body size (mm)	97	<5	5-10	10-20	20-40	>40
feeding behaviour	97	grazer/scraper	shredder	gatherer/collector	filter feeder	predator
locomotion type	91	swimming/scatting	burrowing	sprawling/walking	semisessile	-
respiration type	100	tegument	gills	plastron	aerial	-
life-cycle	90	semivoltine	univoltine	plurivoltine	-	-
dispersal mode	98	aquatic passive	aquatic active	aerial passive	aerial active	-
dispersal via water (m)	78	<10	10-100	100-1000	>1000	-

Table S2: Niche overlap (D), P-value of the niche similarity test (p-D) and three dynamic niche indices (niche expansion, stability and restriction) for winners belonging to four categories: ExFil, expanding fillers; Fill, fillers; Exp, expanders; Shif, shifters. Niche overlap and P-values are in bold for species where niche similarity tests confirmed significant niche conservatism. The last two columns show the shifts in species' centroid positions (in % of gradient length) between periods along the first and second PCA axes. Shifts of >10% of the gradient length on each axis are shown in bold.

Cat.	Species	D	p-D	Expansion	Stability	Restriction	PCA1	PCA2
Fill	*Ancylus fluviatilis*	**0.756**	**0.001**	0.090	0.910	0.005	0.82	-4.42
	Dugesia gonocephala	**0.835**	**0.001**	0.039	0.961	0.028	-3.39	0.31
	Ecdyonurus torrentis	**0.749**	**0.003**	0.093	0.907	0.018	-4.69	-3.42
	Habroleptoides confusa	**0.826**	**0.002**	0.056	0.944	0.038	-0.81	-5.27
	Habrophlebia lauta	**0.809**	**0.001**	0.041	0.959	0.021	0.86	-1.27
	Hydropsyche siltalai	**0.733**	**0.006**	0.109	0.891	0.040	8.30	-1.98
	Ibisia marginata	**0.786**	**0.008**	0.017	0.983	0.055	-2.89	-1.62
	Leuctra hippopus	**0.814**	**0.001**	0.045	0.955	0.032	-0.93	1.62
	Nigrobaetis muticus	**0.800**	**0.002**	0.068	0.932	0.026	-5.78	-0.70
	Polycentropus flavomaculatus	**0.735**	**0.024**	0.044	0.956	0.015	9.48	-1.68
	Propappus volki	**0.728**	**0.015**	0.029	0.971	0.047	1.65	4.67
	Sericostoma personatum/schneiderii	**0.834**	**0.001**	0.014	0.986	0.046	1.62	-0.62
ExFil	*Brachyptera risi*	**0.557**	**0.041**	0.328	0.672	0.004	**-12.25**	2.26
	Centroptilum luteolum	**0.611**	**0.031**	0.176	0.824	0.000	-1.05	0.09
	Eiseniella tetraedra	0.529	0.169	0.053	0.947	0.224	3.26	-1.06
	Ephemera danica	**0.642**	**0.048**	0.169	0.831	0.008	4.42	-4.49
	Mystacides azurea	**0.634**	**0.045**	0.143	0.857	0.034	9.93	0.26
	Odontocerum albicorne	**0.684**	**0.017**	0.026	0.974	0.137	0.53	-3.59
	Orectochilus villosus	**0.682**	**0.019**	0.122	0.878	0.009	**13.05**	-0.69
	Paraleptophlebia submarginata	0.578	0.070	0.255	0.745	0.065	6.58	**-11.06**
	Pisidium casertanum	**0.663**	**0.036**	0.077	0.923	0.071	1.66	0.79
	Pisidium subtruncatum	0.622	0.071	0.256	0.744	0.006	9.06	0.37
	Platambus maculatus	0.510	0.222	0.314	0.686	0.045	**16.15**	0.62
	Sialis fuliginosa	**0.676**	**0.023**	0.119	0.881	0.142	3.67	**-11.49**
	Silo pallipes	**0.718**	**0.013**	0.082	0.918	0.090	1.56	4.70
Exp	*Athripsodes bilineatus*	0.428	0.071	0.545	0.455	0.071	**15.35**	-2.58
	Cheumatopsyche lepida	0.492	0.058	0.538	0.462	0.003	**11.05**	**-13.52**
	Lumbriculus variegatus	0.321	0.207	0.491	0.509	0.047	-1.94	-1.60
	Pisidium personatum	0.341	0.119	0.581	0.419	0.121	-0.92	**14.91**
	Protonemura aestiva/auberti	0.333	0.051	0.560	0.440	0.000	-6.08	5.40
Shif	*Agapetus ochripes*	**0.603**	**0.024**	0.025	0.975	0.214	3.42	9.41
	Allogamus auricollis	**0.762**	**0.004**	0.017	0.983	0.142	0.19	2.53
	Athripsodes cinereus	0.619	0.072	0.118	0.882	0.021	0.16	-0.01
	Calopteryx virgo	0.518	0.120	0.255	0.745	0.125	**14.04**	5.07
	Lepidostoma basale	0.544	0.150	0.096	0.904	0.153	**14.41**	1.23
	Leuctra nigra	0.407	0.191	0.371	0.629	0.308	-0.47	**16.04**
	Micrasema minimum	**0.790**	**0.003**	0.148	0.852	0.037	-3.47	2.62
	Nais alpina	0.443	0.231	0.236	0.764	0.196	**-15.22**	3.65
	Nemoura cinerea	0.611	0.070	0.115	0.885	0.174	**10.72**	-2.24
	Nigrobaetis niger	0.525	0.149	0.223	0.777	0.180	**10.93**	-5.71
	Oecismus monedula	**0.702**	**0.011**	0.222	0.778	0.145	-2.07	-3.37
	Oulimnius tuberculatus	0.457	0.217	0.194	0.806	0.152	**-23.00**	-3.13
	Philopotamus montanus	0.403	0.212	0.096	0.904	0.359	-3.79	2.51

Table S3: Descriptive statistics of the used environmental variables, separately for each period. Transformation type: none, no transformation; sqrt, square root; log, logarithmic.

**Environmental variables used in niche consideration**	**Transfor-mation**	**Initial period**			**Final period**		
		**Median**	**Mean**	**SD**	**Median**	**Mean**	**SD**
Annual precipitation [mm]	none	65.5	70.7	14.6	65.6	65.6	9.1
Phi [-]	sqrt	1.712	1.780	0.324	1.844	1.885	0.384
NO3-N [mg/l]	log	0.374	0.354	0.478	0.220	0.219	0.503
Riffles share [%]	none	60.0	58.5	20.9	80.0	68.2	24.3
Forest area [km2]	log	2.389	2.149	0.854	2.416	2.184	0.843
Unfavourable surf. area [%]	log	1.459	1.688	1.258	1.099	1.452	1.207
Total P [mg/l]	log	-3.058	-2.921	0.768	-2.938	-2.864	0.481
BOD5 [mg/l]	sqrt	1.063	1.147	0.243	1.140	1.192	0.229
NH4-N [mg/l]	log	-3.244	-3.142	0.684	-3.244	-3.232	0.419
**Only passively correlated**							
Mean January temp. (°C)	none	-3.36	-2.96	1.02	-0.43	-0.56	0.50
Mean July temp. (°C)	none	16.38	16.35	0.90	18.96	18.86	0.88

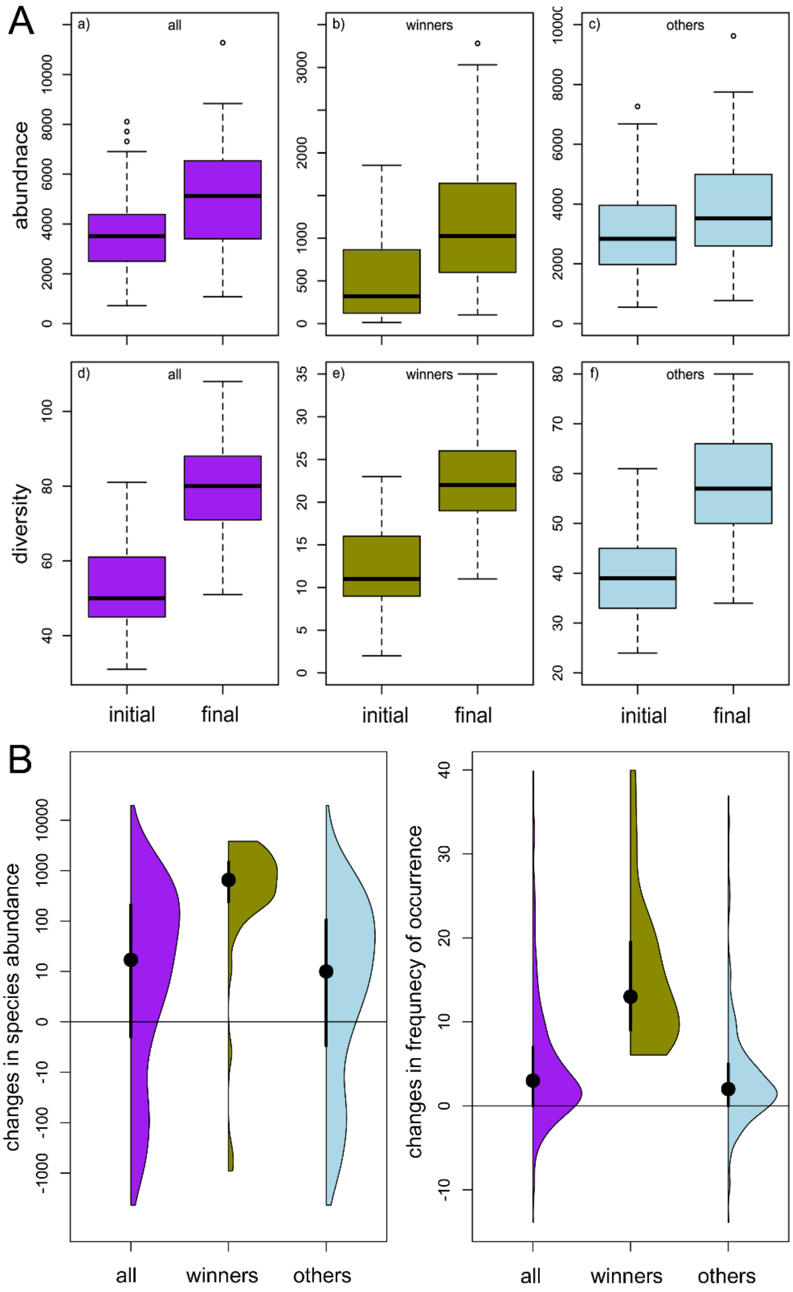

Figure S1: **A**, total abundance (i.e., summed across all species) and taxa richness at the 65 study sites in the initial and final periods calculated for (a) all taxa, (b) winner species only and (c) other taxa only. For details on boxplots see the caption for Fig. 4. **B**, variation in changes of species abundance (summed across all sites) and occurrence showed across all species, winners only and other species only. The change is calculated as a difference between species overall abundance/occurrence in the final and initial periods. Points refer to median values, bold lines mean interquartile ranges and thin lines ranges. Areas represent smoothen density distribution.

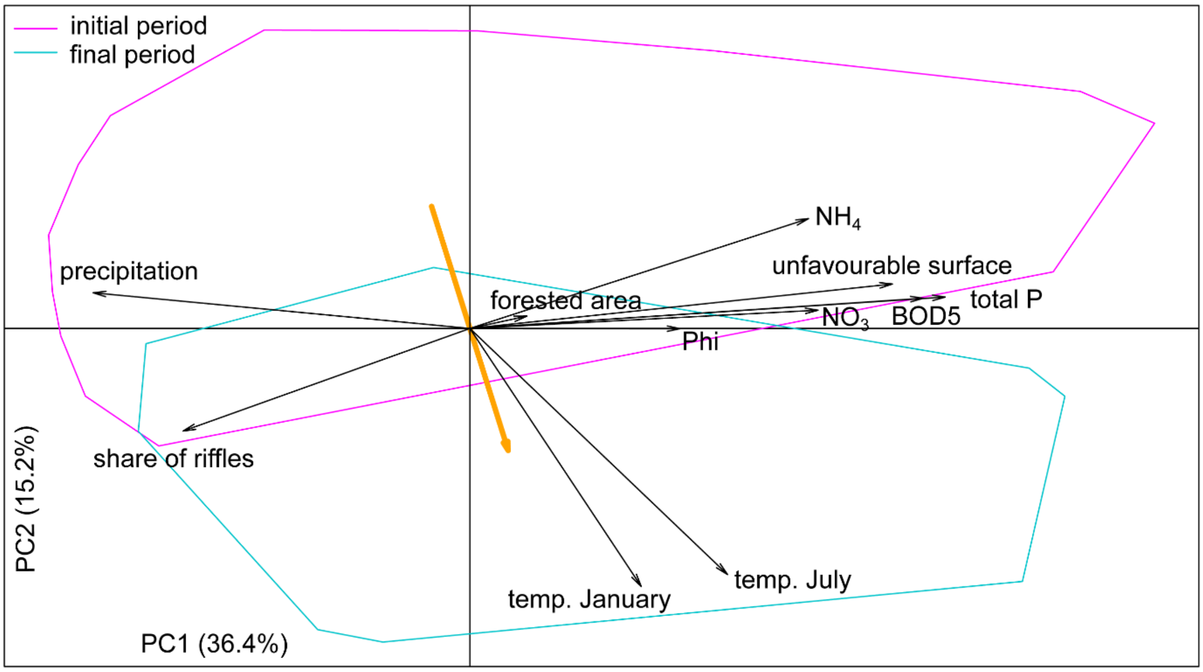

Figure S2: Available environmental spaces in initial and final periods expressed by the first two PCA axes based on all environmental variables including air temperature data.

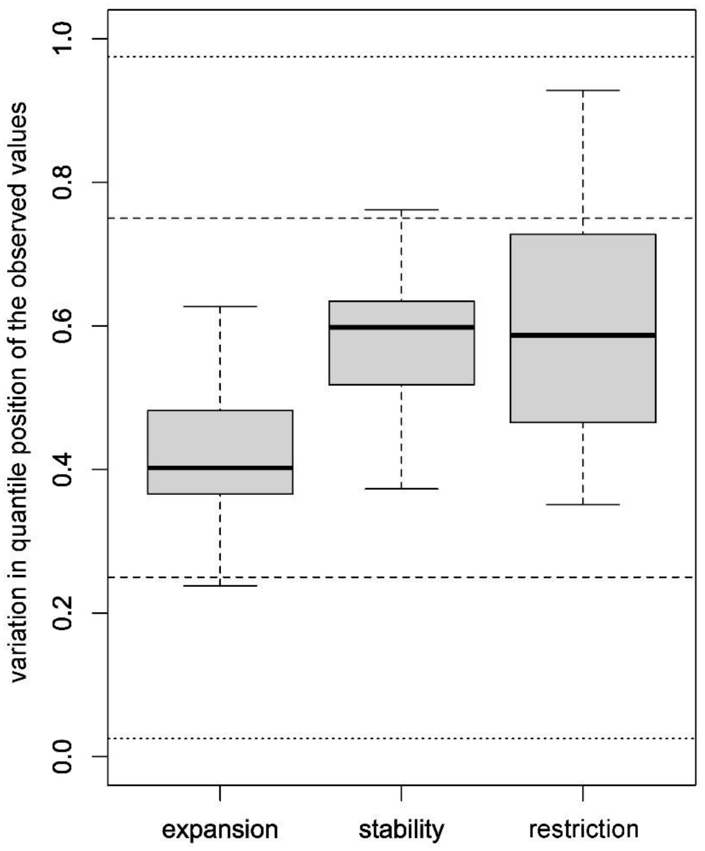

Figure S3: Position of the original observed value of three niche dynamic indexes (niche expansion, stability and restriction) within a range of 1000 values obtained by leave-one-out jack-knife procedure. Dotted lines represent 95% reference range; while dashed lines represent interquartile range.

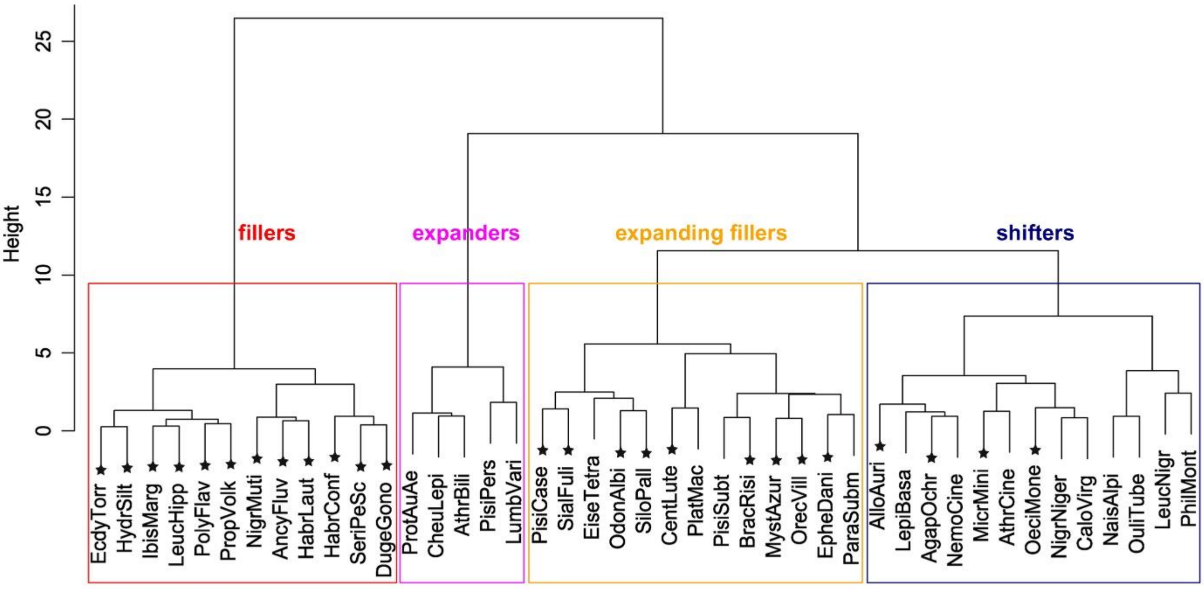

Figure S4: Dendrogram visualising the results of cluster analysis of six niche-related parameters (Ward's method, k = 4), resulting in four winner categories marked by coloured rectangles. Species with significant similarity test (Table S2) are marked by an asterisk on their branch tips.

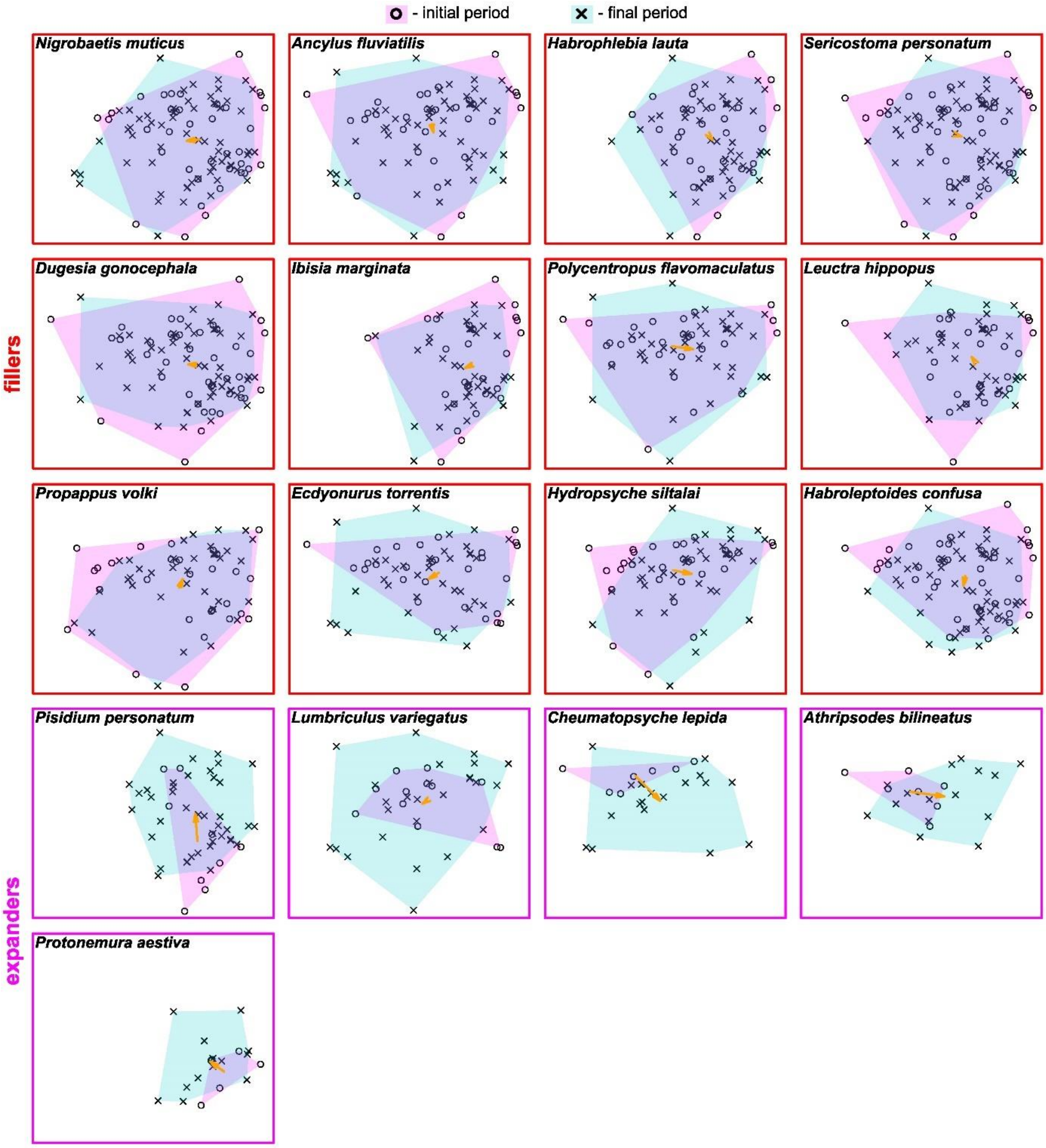

Figure S5a: Environmental niche shifts from initial to final period for winner species classified as fillers and expanders (see Table S2, Fig. S3). Orange arrow shows centroid shift. See Fig. 2 for further details on environmental niche space and main environmental gradients.

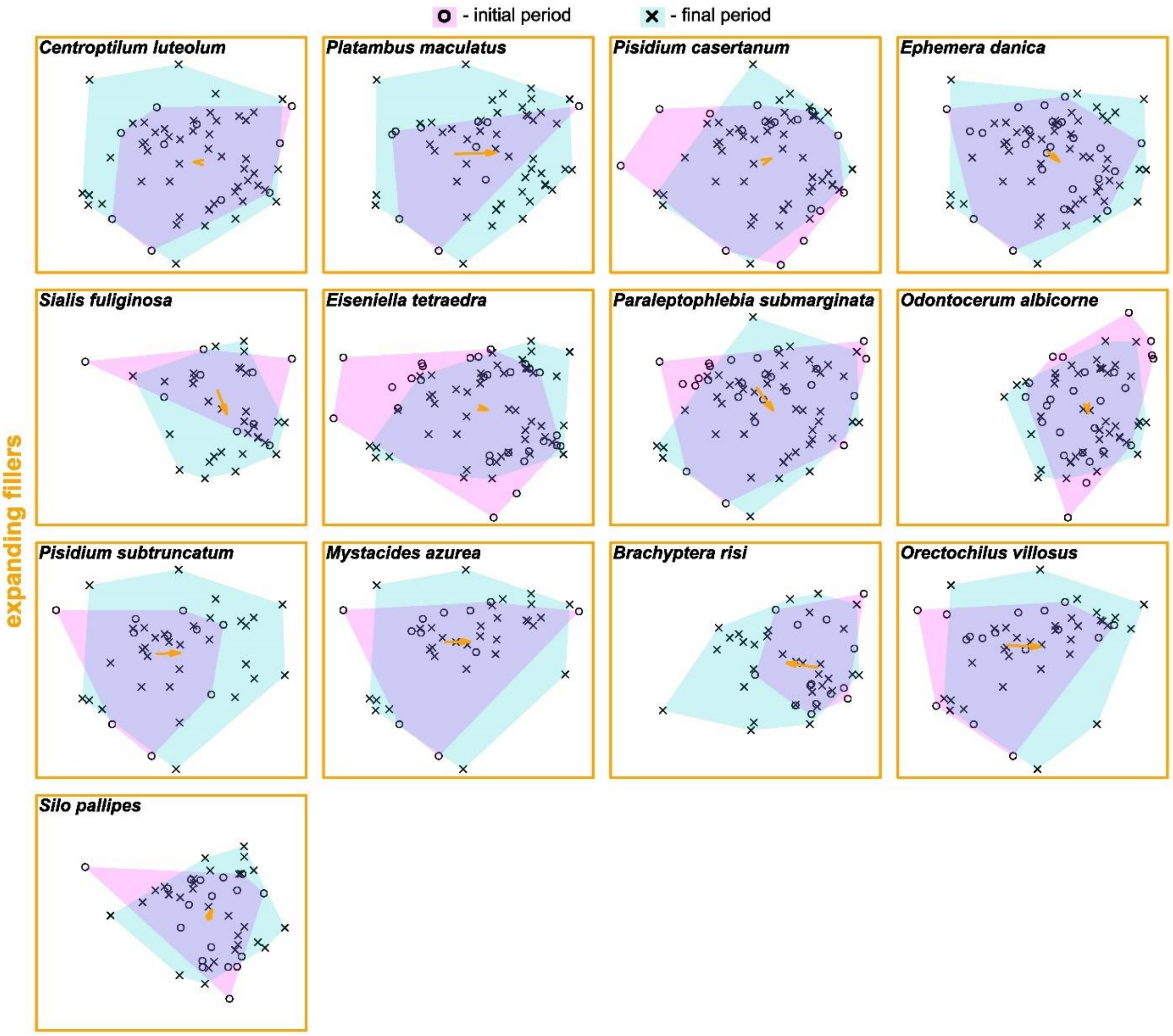

Figure S5b: Environmental niche shifts from initial to final period for winner species classified as expanding fillers (see Table S2, Fig. S3). Orange arrow shows centroid shift. See Fig. 2 for further details on environmental niche space and main environmental gradients.

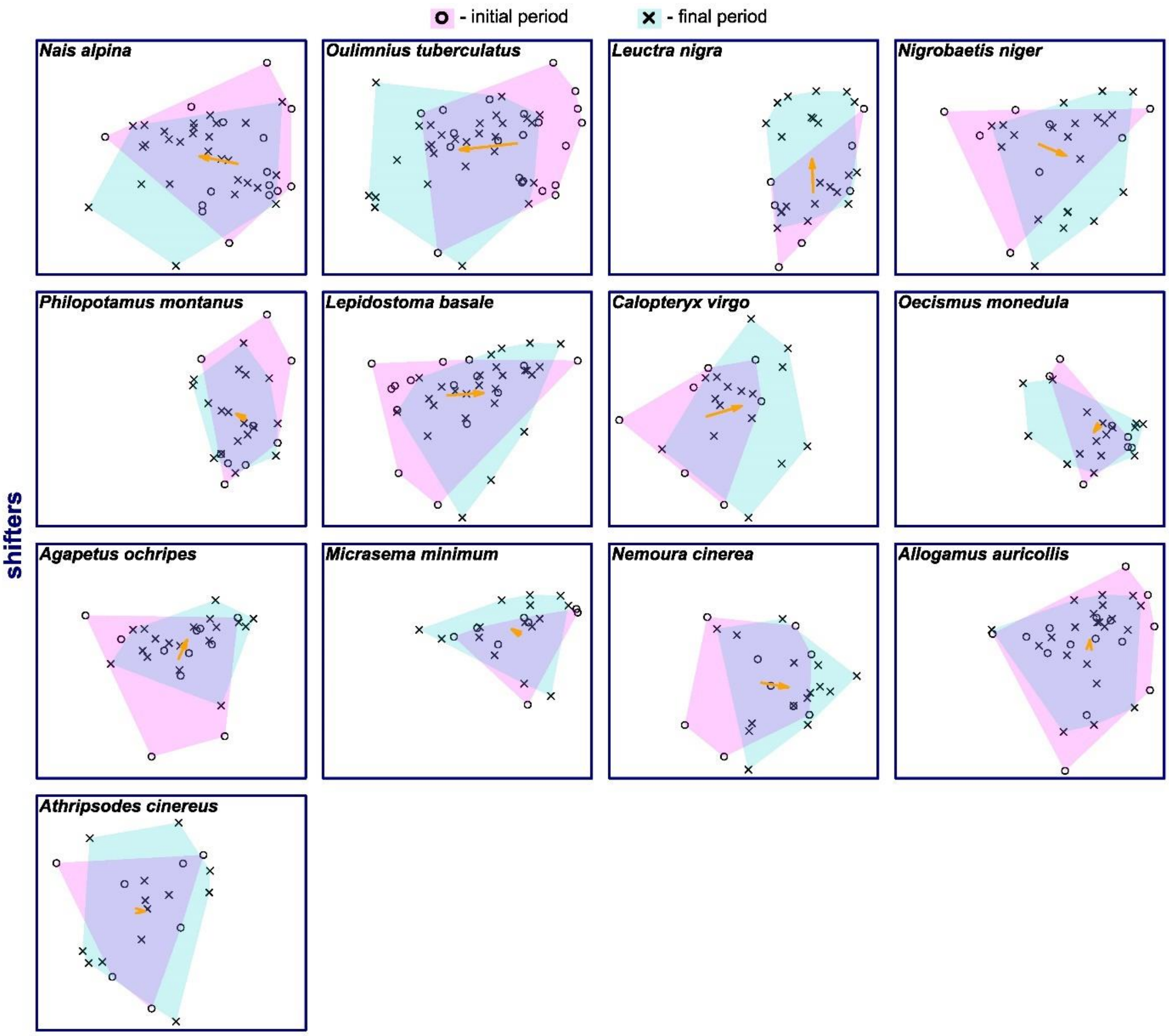

Figure S5c: Environmental niche shifts from initial to final period for winner species classified as shifters (see Table S2, Fig. S3). Orange arrow shows centroid shift. See Fig. 2 for further details on environmental niche space and main environmental gradients.
